# Whole-genome characterization and zoonotic insights of feline rotavirus A genotypes G3P[9] and novel G6P[9] circulating in domestic cats in Thailand

**DOI:** 10.14202/vetworld.2026.81-96

**Published:** 2026-01-08

**Authors:** Yu Nandi Thaw, Kamonpan Charoenkul, Chanakarn Nasamran, Ekkapat Chamsai, Waleemas Jairak, Eaint Min Phyu, Hnin Wai Phyu, Supassama Chaiyawong, Somsak Pakpinyo, Alongkorn Amonsin

**Affiliations:** 1 Department of Veterinary Public Health, Faculty of Veterinary Science, Chulalongkorn University, Bangkok, Thailand; 2 Center of Excellence for Emerging and Re-emerging Infectious Diseases in Animals, Faculty of Veterinary Science, Chulalongkorn University, Bangkok, Thailand

**Keywords:** cats, feline rotavirus A, genetic characterization, genotype G3P[9], genotype G6P[9], Thailand, whole-genome sequencing, zoonotic transmission

## Abstract

**Background and Aim:**

Rotavirus A (RVA) is an enteric pathogen affecting both humans and animals, known for its zoonotic potential. Feline RVA (FeRVA) infections are increasingly reported worldwide; however, data remain limited in Thailand. This study aimed to determine the prevalence, genotype distribution, and whole-genome features of FeRVA found in domestic cats in Thailand, as well as to assess the potential for cross-species transmission.

**Materials and Methods:**

A cross-sectional survey was conducted from January 2022 to December 2023 in Bangkok and nearby provinces. Rectal swab samples (n = 636) were collected from both symptomatic and asymptomatic cats and screened for RVA using reverse-transcription polymerase chain reaction (RT-PCR) targeting the nonstructural protein 5 (NSP5) gene. Samples positive for FeRVA were subjected to whole-genome sequencing (WGS) using Oxford Nanopore technology. Genotypes were assigned based on all 11 gene segments, and phylogenetic analyses were performed using the neighbor-joining method to compare Thai strains with global RVA reference strains.

**Results:**

The FeRVA positivity rate was 1.41% (9/636). Three FeRVA-positive samples were successfully sequenced. Whole-genome analysis identified one strain as genotype G3P[9] and two strains as genotype G6P[9]. The G6P[9] strains showed the genetic constellation G6-P[9]-I2-R2-C2-M2-A3-N2-T3-E3-H3, identical to feline and human RVA G6P[9] strains previously reported in Japan. The G3P[9] strain displayed high nucleotide identity with Thai and East Asian human RVAs. Most FeRVA-positive cats were asymptomatic, and no significant association was found between infection status and age, season, or clinical signs. Analysis of the viral protein 7 antigenic regions revealed conserved amino acids, apart from a single substitution (S90P) in G6P[9].

**Conclusion:**

This study reports the first detection of the novel FeRVA genotype G6P[9] in Thailand and provides comprehensive genomic evidence of FeRVA diversity in domestic cats. The close genetic relationship between Thai-FeRVA strains and human RVA strains highlights the potential for interspecies transmission. Enhanced surveillance and One Health–based monitoring are recommended to improve early detection and prevent zoonotic spread.

## INTRODUCTION

Rotavirus (RV), a member of the family Reoviridae, is classified into nine species (A, B, C, D, F, G, H, I, and J) [1]. Rotavirus A (RVA) is the most clinically significant species and a major cause of severe diarrhea in humans and animals worldwide. The RVA genome contains 11 double-stranded RNA segments that encode six structural viral proteins (VP1–VP4, VP6, and VP7) and five nonstructural proteins (NSP1–NSP5). Two main classification systems are used to determine the genetic diversity of RVA. The first system is based on the outer capsid proteins VP7 and VP4, which include neutralization antigens and define the G and [P] genotypes, respectively [2]. The second system classifies RVA based on the genotypes of all 11 genome segments (VP7-VP4-VP6-VP1-VP2-VP3-NSP1-NSP2-NSP3-NSP4-NSP5), summarized as the genotype constellation Gx-P[x]-Ix-Rx-Cx-Mx-Ax-Nx-Tx-Ex-Hx, as established by the RV Classification Working Group (RCWG). This full-genome genotyping approach aids in studying viral evolution, cross-species transmission, and reassortment events between human and animal RVAs. To date, more than 41 G genotypes and 57 [P] genotypes have been documented across humans and various animal hosts (https://rega.kuleuven.be/cev/viralmetagenomics/virus-classification/rcwg). Although many RVA strains demonstrate host specificity, several genotypes can infect multiple species, indicating the potential for interspecies transmission [3, 4].

Feline RVA (FeRVA) was first identified in cats in 1978 through serological testing. The genotype G3P[3] remains the most common FeRVA genotype reported worldwide [5]. Additionally, genotypes G3P[9] and G6P[9] have been more recently detected in cats [5–7]. Although FeRVA infections in cats are usually asymptomatic or only cause mild illness, they present a recognized zoonotic risk, with documented human infections linked to feline-origin strains [8–10]. Several studies indicate that human G3P[3], G3P[9], and G6P[9] strains may have resulted from direct cross-species transmission from cats, with such events reported in United Kingdom [5], Japan [6, 9], South Korea [10], Italy [11], Israel [12], and Tunisia [13].

Information on FeRVA in Thailand remains limited. Previous studies have identified FeRVA genotypes G3P[3] and G3P[9] in domestic cats and have emphasized their zoonotic potential, particularly regarding possible transmission to humans [7, 14].

Although FeRVA has been documented in several countries and across multiple genotypes, including G3P[3], G3P[9], and the emerging G6P[9], its epidemiology in Thailand remains poorly understood. Few studies have examined FeRVA in domestic cats, and existing data are limited to partial genotyping or small-scale surveillance, resulting in an incomplete picture of circulating strains, their genetic diversity, and their zoonotic potential. Importantly, there is a lack of whole-genome sequencing (WGS) analyses of FeRVA in Thailand, despite increasing international reports of feline- and human-derived G3P[9] and G6P[9] strains that indicate ongoing cross-species transmission. The absence of comprehensive genomic data hampers efforts to trace viral origins, detect reassortment events, or compare local feline strains with regional human RVA strains. Consequently, a significant knowledge gap exists regarding the prevalence, molecular features, and evolutionary patterns of FeRVA in Thai domestic cats, along with their potential public health implications within a One Health approach.

This study aimed to fill these gaps by conducting a cross-sectional investigation of FeRVA infections in domestic cats in Thailand through a combination of molecular detection, genotyping, and whole-genome analysis. Specifically, it aimed to determine the prevalence of FeRVA among both symptomatic and asymptomatic cats, identify circulating G and P genotypes, and analyze the complete genetic makeup of detected strains using WGS. Phylogenetic and comparative genomic analyses were performed to explore the evolutionary relationships between Thai-FeRVA strains and reference RVA strains from various hosts and regions. By combining epidemiological and genomic data, this study seeks to provide the first detailed whole-genome insights into FeRVA diversity in Thailand, assess the potential for interspecies transmission between cats and humans, and support improved surveillance and One Health–based monitoring strategies.

## MATERIALS AND METHODS

### Ethical approval

This study was conducted in strict accordance with the ethical standards and animal welfare regulations of Chulalongkorn University. All procedures involving animals were reviewed and approved by the Chulalongkorn University Animal Care and Use Committee (CU-VET IACUC) under protocol numbers CU-VET IACUC# 2031035 and CU-VET IACUC# 2331098. These protocols ensure compliance with institutional guidelines, the Animal Care and Use of Laboratory Animals framework, and the international principles outlined in the Animal Research: Reporting of *In Vivo* Experiments (ARRIVE) guidelines.

All cats included in the study were privately owned and brought to participating veterinary hospitals for routine clinical care or medical consultation. Only non-invasive sampling procedures were performed. Rectal swab collection was carried out by licensed veterinarians or trained personnel under the supervision of a veterinarian, ensuring minimal discomfort and no harm to the animals. Cats with severe cardiac conditions or at risk of mortality were excluded to prevent undue stress or deterioration of their clinical status.

Before sampling, veterinarians explained the study goals, sampling methods, and data confidentiality to the owners. Verbal informed consent was obtained from all cat owners, in line with the ethical standards of CU-VET IACUC and the Animal Research: Reporting of In Vivo Experiments 2.0 guidelines for non-invasive surveillance studies. No sedation, restraint beyond routine clinical handling, or experimental procedures were used. All animals were managed according to standard veterinary care practices, with their welfare prioritized throughout sample collection, storage, and transportation.

All biological samples were handled following biosafety level 2 protocols, and personal protective equipment was worn throughout sample collection to ensure the safety of personnel and animals. Data collected during the study were anonymized to protect the owner and animal identities.

### Study period and location

This study was carried out from January 2022 to December 2023 at eight private animal hospitals in Bangkok, Nonthaburi, and Samut Prakan, Thailand.

### Study design and sample collection

A cross-sectional survey of FeRVA was conducted. Animal hospitals were selected based on convenient locations and the cooperation of hospital staff. A total of 636 rectal swab samples were collected from cats with either asymptomatic or symptomatic conditions, including vomiting and diarrhea. Cats brought to animal hospitals with owner registration were included. Animals with pre-existing cardiac conditions and those in a dying state were excluded from the study. All hospitalized cats were eligible for sampling. Sampling was performed randomly among hospitalized cats, with a focus on those exhibiting gastrointestinal clinical signs. There were no restrictions regarding age, sex, or breed. Demographic data, including age, sex, breed, and clinical signs, were recorded during sampling.

### Sample collection procedure

Cotton swabs were carefully inserted into the rectum and rotated to collect rectal swab samples. The collected swabs were placed in viral transport media (Eagle Minimum Essential Medium) and stored at 4°C, then transported to the laboratory within 24 h. Safety precautions and PPE were strictly followed during sample collection.

### RNA extraction and FeRVA detection

RNA was extracted with the GeneAll® GENTiTM Viral DNA/RNA Extraction Kit (GeneAll®; Lisbon, Portugal) using a GENTiTM 32 (GeneAll®; Lisbon, Portugal) according to the manufacturer’s instructions. NSP5-specific primers were used to detect FeRVA, as previously described [15]. To identify FeRVA, RNA was screened by one-step reverse-transcription polymerase chain reaction (RT-PCR) with specific primers using SuperScript™ III RT-PCR with Platinum™ Taq Mix (Invitrogen, Thermo Fisher Scientific, USA). Briefly, one-step RT-PCR was performed in a final volume of 25 μL containing 3 μL of template RNA, 12.5 μL of 2X Reaction Mix, 0.5 μL of 1 μM forward and reverse primers, 1 μL of SuperScript III RT (Invitrogen, Thermo Fisher Scientific, USA), and distilled water. The assay included a cDNA synthesis step at 55°C for 15 min and 94°C for 2 min, followed by 40 cycles at 94°C for 15 s, 52°C for 30 s, 68°C for 45 s, and a final extension at 68°C for 5 min. The PCR product was electrophoresed on a 1.5% agarose gel and stained with RedSafeTM (iNtRON Biotechnology, Inc., Korea) at 100 V for 45 min. The expected size of the RVA-positive amplified products was 208 bp.

### WGS of FeRVA

Positive FeRVA (n = 3) were selected and subjected to WGS. The criteria for selecting RVAs were based on high-quality RNA, location, and collection date. RNA quality and quantity were measured using a NanoDrop spectrophotometer (concentration >20 ng/μL and 260/280 ratio >2.0). This approach ensured that only high-quality RNA suitable for accurate and reliable genome sequencing was used. WGS was conducted by amplifying each gene using oligonucleotide primer sets as previously described [16, 17], with new primer sets designed using the Primer3Plus program (Supplement Table 1). Briefly, nucleotide amplification for each gene was conducted by one-step RT-PCR in a final total volume of 25 μL, consisting of 3 μL of template RNA, 12.5 μL of 2X Reaction Mix, 0.5 μL of 10 μM forward and reverse primers, 1 μL of SuperScript III RT (Invitrogen, CA), and distilled water. RT-PCR conditions included a cDNA synthesis step at 55°C for 30 min, an initial denaturation at 94°C for 2 min, followed by 40 cycles of denaturation at 94°C for 30 s, annealing at 45°C–53°C for 30 s, extension at 68°C for 1–4 min, and a final extension at 68°C for 5–7 min. Agarose gel electrophoresis was performed to confirm positive PCR amplification.

The PCR products of each gene were pooled and sequenced using the Oxford Nanopore MinION device (MinION Mk1b) and MinION flow cells (FLO-FLG114, R10.4.1) with the Rapid Sequencing Kit (SQK-RAD114) (ONT, UK). The DNA library and flow cell priming mix were prepared per the manufacturer’s instructions. To prepare the DNA library, 5 μL of pooled PCR product from each gene was mixed with 0.5 μL of fragmentation mix (FRA) and incubated at 30°C for 2 minutes, then at 80°C for 2 min. After cooling on ice, 0.5 μL of the rapid adapter (RAP) was added, and the mixture was incubated at 25°C for 5 min. Then, 15 μL of sequencing buffer (SQB) and 10 μl of loading beads (LB) were added to complete the DNA library loading. The flow cell priming mix was prepared by combining 3 μL of flow cell tether with 117 μL of flow cell flush. To start sequencing, 120 μL of the flow cell priming mix was added to the flow cell, followed by an additional 30 μL of sequencing mix, 5 μL of the prepared DNA library, 10 μL of library beads (LB), and 15 μL of sequencing buffer. The sequencing process was initiated using MinKNOW (version 24.11.8; ONT, UK). Sequence reading and base calling, i.e., converting the electrical signals (fast5 files) into nucleotide sequences (fastq files), were performed with the GPU-enabled Guppy basecaller (version 6.5.7). A minimum quality Score (Qscore) of 7 was used to exclude low-quality sequences from the BCP. The nucleotide sequences were assembled and analyzed with Racon software (version 0.5.0). The sequences of each RVA gene segment were retrieved in FASTA format and compared to the NCBI database using BLAST to identify the closest matching reference sequences. Finally, the consensus sequences of each virus gene segment were exported as FASTA files for further analysis.

### Phylogenetic and genetic analysis of FeRVA

The whole-genome sequences of FeRVA were phylogenetically analyzed by comparing the nucleotide sequences of each gene with those of other reference RVAs from different genotypes available in the GenBank database, representing various geographical origins and host species. Phylogenetic trees of whole-genome sequences were constructed using MEGA v.11.0 (Tempe, AZ, USA) with 1,000 bootstrap replicates, employing the neighbor-joining method and the Kimura 2-parameter model. A pairwise comparison of Thai-FeRVA nucleotides and amino acids was performed using reference RVAs from the same and different genotypes from the GenBank database. Genetic analysis focused on the complete VP7 amino acid sequences by comparing the major antigenic regions (A, B, C, and F) of Thai-FeRVAs with those of reference RVAs from various genotypes. Alignment was conducted using MegAlign software v.5.03 (DNASTAR Inc., Madison, WI, USA). Variable and unique amino acids related to the major neutralizing antigens of the viruses were evaluated. The genetic constellation of RVA was determined by analyzing the combination of genotypes across all 11 gene segments (*VP7, VP4, VP6, VP1, VP2, VP3, NSP1, NSP2, NSP3, NSP4*, and *NSP5*), following the RCWG system. The genetic constellation of Thai-FeRVAs was compared with that of reference RVAs. Reference RVAs from various species, including dogs, cats, humans, cattle, and bats, were included in the analysis.

### Statistical analysis

The association between FeRVA occurrence in domestic cats and demographic factors such as animal age, clinical status, and season was analyzed using the chi-square test (SPSS Statistics, version 29.0.1.0, IBM Corp., NY, USA). A p-value of <0.05 was considered statistically significant.

## RESULTS

### Prevalence and demographic association of FeRVA

The overall occurrence of FeRVA positivity in the cats was 1.41% (9/636) (Table 1 and Supplementary Table 2). FeRVA was detected throughout the year; however, the virus was most common during winter (November–January). Infection was observed in both symptomatic and asymptomatic animals, with a higher rate in asymptomatic cats. FeRVA was most frequently found in young cats (up to 6 months old). No significant link was found between FeRVA positivity and the cats’ age, season, or clinical status (Table 2).

**Table 1:** Sample collection and feline rotavirus A detection in cats.

Year	Month/Year	Collected Sample	No. of positive samples (%)
2022	Jan-22	4	0 (-)
	Feb-22	8	0 (-)
	Mar-22	11	0 (-)
	Apr-22	5	0 (-)
	May-22	10	0 (-)
	Jun-22	14	0 (-)
	Jul-22	16	0 (-)
	Aug-22	4	0 (-)
	Sep-22	4	0 (-)
	Oct-22	10	0 (-)
	Nov-22	28	1 (3.57)
	Dec-22	57	4 (7.02)
Total		171	5 (2.92)
2023	Jan-23	44	0 (-)
	Feb-23	42	0 (-)
	Mar-23	70	1 (1.43)
	Apr-23	21	1 (4.76)
	May-23	31	0 (-)
	Jun-23	39	0 (-)
	Jul-23	28	0 (-)
	Aug-23	42	1 (2.38)
	Sep-23	33	0 (-)
	Oct-23	26	0 (-)
	Nov-23	54	0 (-)
	Dec-23	35	1 (2.86)
Total		465	4 (0.86)
Grand Total		636	9 (1.41)

**Table 2:** FeRVA occurrence by age, clinical status, and season.

Factors	FeRVA			

	Positive (%)	Negative (%)	χ²	p-value
Age				
Up to 6 ms	4 (2.38)	164 (97.62)	2.144	0.349
Older than 6 months to 2 years	2 (1.08)	184 (98.92)		
More than 2 years	2 (0.80)	247 (99.20)		
Unknown	1 (3.03)	32 (96.97)		
	9	627		
Clinical Status				
Asymptomatic	7 (2.72)	250 (97.28)	3.362	0.183
Symptomatic	2 (0.62)	321 (99.38)		
Unknown status (N/A)	0 (0.00)	56 (100.00)		
	9	627		
Season				
Winter (November–January)	6 (2.71)	215 (97.29)	4.377	0.117
Summer (February– May)	2 (1.00)	198 (99.00)		
Rainy (June–October)	1 (0.47)	214 (99.53)		
	9	627		

* Statistical significance = p < 0.05, FeRVA = Feline rotavirus A.

### WGS and phylogenetic analysis

**Figure 1:**
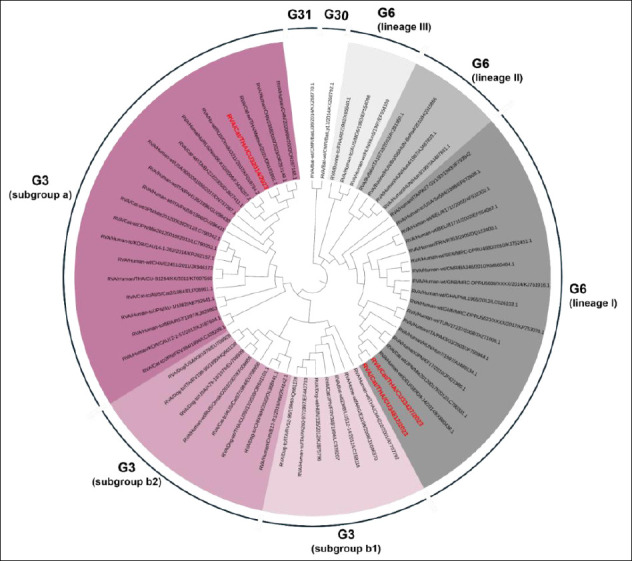
Phylogenetic tree of the complete *VP7* gene of Thai-feline rotavirus A (FeRVA) using the Kimura-2 model with 1,000 bootstrapping replicates. The red color represents the Thai-FeRVAs characterized in this study.

**Figure 2:**
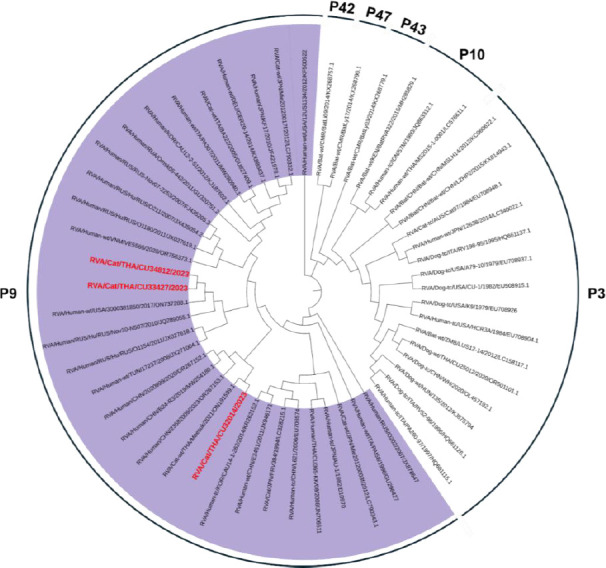
Phylogenetic tree of the complete *VP4* gene of Thai-feline rotavirus A (FeRVA) using the Kimura-2 model with 1,000 bootstrapping replicates. The red color represents the Thai-FeRVAs characterized in this study.

Three FeRVA-positive samples (CU32014, CU33427, and CU34812) were successfully sequenced for WGS (Table 3). The WGS data of Thai-FeRVAs were submitted and are available in the GenBank database under accession numbers PV650015-47 (Supplementary Table 3). Phylogenetic analysis of the complete *VP7* and *VP4* genes revealed that one FeRVA (CU32014) was classified as G3P[9], while two FeRVAs (CU33427, CU34812) were classified as G6P[9]. Thai-FeRVA G6P[9], a novel genotype, was identified and has not been reported in Thailand before. Phylogenetic analysis of the VP7 gene showed that the Thai-FeRVA G3P[9] (CU32014) belonged to the G3 subgroup “a” and was closely related to previously reported Thai-FeRVA G3P[9] and human RVAs from China. The two Thai-FeRVA G6P[9] (CU33427, CU34812) belonged to G6 lineage I and were closely related to feline and human RVAs from Japan (Figure 1). For the *VP4* gene, all Thai-FeRVAs grouped into the P9 genotype and were closely related to human RVAs from China, Russia, Tunisia, and the United States (Figure 2). Phylogenetic analyses of other structural proteins (VP) and NSP are provided in Supplementary Figures 1–3.

**Table 3:** Detailed descriptions of the FeRVA characterized in this study.

ID	Year	Location	Age	Sex	Breed	Clinical Sign	Genotype	Gene	Accession No.
FeRVA									
CU32014	Mar-23	Bangkok	1yr	NM	DSH	Healthy	G3P[9]	WGS	PV650015-25
CU33427	Aug-23	Bangkok	3mth	M	Thai	Diarrhea	G6P[9]	WGS	PV650026-36
CU34812	Dec-23	Bangkok	5mth	F	DSH	Healthy	G6P[9]	WGS	PV650037-47

### Genetic constellation of the Thai-FeRVA

In this study, the genetic makeup of Thai-FeRVA G6P[9] was identified as G6-P[9]-I2-R2-C2-M2-A3-N2-T3-E3-H3, which matched the feline RVAs G6P[9] from Japan (JPN/Mie/20120017/2012) and human RVAs from Japan (JPN/KF17/2010). Thai-FeRVA G3P[9] showed a G3-P[9]-I2-R2-C2-M2-A3-N2-T3-E3-H3 genotype, identical to Thai-FeRVA G3P[9] (THA/Meesuk/2021) and human RVAs from China (CHN/2020999/2020; CHN/23582009/2023) and Korea (KOR/CAU12-2-51/2013) (Table 4).

**Table 4:** Genetic constellation of FeRVAs and reference RVAs from dogs, cats, cattle, bats, and humans.

Virus	Strain	Year	Country	Gene										

				*VP7*	*VP4*	*VP6*	*VP1*	*VP2*	*VP3*	*NSP1*	*NSP2*	*NSP3*	*NSP4*	*NSP5*
This study														
RVA/Cat/THA/CU33427/2023/ G6P[9]	CU33427	2023	Thailand	G6	P9	I2	R2	C2	M2	A3	N2	T3	E3	H3
RVA/Cat/THA/CU34812/2023/ G6P[9]	CU34812	2023	Thailand	G6	P9	I2	R2	C2	M2	A3	N2	T3	E3	H3
RVA/Cat/THA/CU32014/2023/ G3P[9]	CU32014	2023	Thailand	G3	P9	I2	R2	C2	M2	A3	N2	T3	E3	H3
Reference strains														
Feline														
RVA/Cat-wt/THA/Meesuk/2021/ G3P[9]	Meesuk	2021	Thailand	G3	P9	I2	R2	C2	M2	A3	N2	T3	E3	H3
RVA/Cat-wt/ITA/BA222/2005/ G3P[9]	BA222	2005	Italy	G3	P9	I2	R2	C2	M2	A3	N1	T3	E2	H3
RVA/Cat-tc/AUS/Cat2/1984/ G3P[9]	Cat2	1984	Australia	G3	P9	I3	R3	C3	M3	A3	N1	T6	E3	H3
RVA/Cat/THA/RV25045/2020/ G3P[9]	RV25045	2020	Thailand	G3	P3	I8	R3	C3	M3	A9	N3	T3	E3	H6
RVA/Cat/JPN/FRV317/1994/ G3P[9]	FRV317	1994	Japan	G3	P9	I3	R3	C3	M3	A3	N3	T3	E3	H6
RVA/Cat/JPN/Mie20120003f/2012/G3P[9]	Mie20120003f	2012	Japan	G3	P9	I3	R3	C3	M3	A3	N3	T3	E3	H3
RVA/Cat/JPN/Mie20120017f/2012/ G6P[9]	Mie20120017f	2012	Japan	G6	P9	I2	R2	C2	M2	A3	N2	T3	E3	H3
Canine														
RVA/Dog/THA/CU132/2017/ G3P[3]	CU132	2017	Thailand	G3	P3	I3	R3	C3	M3	A9	N2	T3	E3	H6
RVA/Dog/CU20139/2017/ G3P[3]	CU20139	2019	Thailand	G3	P3	I3	R3	C3	M3	A9	N2	T3	E3	H6
RVA/THA/CU23379/2019/ G3P[3]	CU23379	2020	Thailand	G3	P3	I3	R3	C3	M3	A9	N2	T3	E3	H6
RVA/Dog/THA/RV25012/2020/ G3P[3]	RV25012	2020	Thailand	G3	P3	I3	R3	C3	M3	A9	N2	T3	E3	H6
RVA/Dog/THA/DC25170/2020/ G3P[3]	DC25170	2020	Thailand	G3	P3	I3	X	C3	M3	A9	N2	T3	E3	H6
RVA/Dog-tc/CHN/WH/2020/ G3P[3]	WH	2020	China	G3	P3	X	X	X	X	X	X	X	X	X
Human														
RVA/Human-wt/CHN/2020999/2020/ G3P[9]	2020999	2020	China	G3	P9	I2	R2	C2	M2	A3	N2	T3	E3	H3
RVA/Human-wt/CHN/23582009/2023/ G3P[9]	23582009	2023	China	G3	P9	I2	R2	C2	M2	A3	N2	T3	E3	H3
RVA/Human-wt/CHN/E2451/2011/ G3P[9]	E2451	2011	China	G3	P9	I3	R3	C3	M3	A3	N3	T3	E3	H6
RVA/Human-tc/KOR/CAU12-2-51/2013/ G3P[9]	CAU12-2-51	2013	Korea	G3	P9	I2	R2	C2	M2	A3	N2	T3	E3	H3
RVA/Human-tc/KOR/CAU14-1-262/2014/ G3P[9]	CAU14-1-262	2014	Korea	G3	P9	I3	R3	C3	M3	A3	N3	T1	E3	H6
RVA/Human-wt/ITA/PA158/1996/G3P9	PA158	1996	Italy	G3	P9	I2	R2	C2	M2	A3	N2	T6	E2	H3
RVA/Human-wt/ITA/PAH136/1996/ G3P[9]	PAH136	1996	Italy	G3	P9	I2	R2	C2	M2	A3	N2	T6	E2	H3
Virus	Strain	Year	Country	Gene										
				*VP7*	*VP4*	*VP6*	*VP1*	*VP2*	*VP3*	*NSP1*	*NSP2*	*NSP3*	*NSP4*	*NSP5*
RVA/Hu/RUS/Nov-K10/2006/ G3P[9]	Nov-K10	2006	Russia	G3	P9	I2	X	X	X	X	X	X	X	X
RVA/Human/RUS/Omsk08-42/2008/ G3P[9]	Omsk08-42	2008	Russia	G3	P9	I2	X	X	X	X	X	X	X	X
RVA/Hu-wt/RUS/Omsk/O211/2007/ G3P[9]	Omsk/0211	2007	Russia	G3	P9	I2	X	X	X	X	N2	X	X	H3
RVA/Human-wt/USA/3000381850/2017/ G3P[9]	3000381850	2017	USA	G3	P9	I2	R2	C2	M2	A3	N2	T3	E2	H3
RVA/Human-tc/JPN/AU-1/1982/ G3P[9]	AU-1	1982	Japan	G3	P9	I3	R3	C3	M3	A3	N3	T3	E3	H3
RVA/Human-wt/JPN/KF17/2010/ G6P[9]	KF17	2010	Japan	G6	P9	I2	R2	C2	M2	A3	N2	T3	E3	H3
RVA/Human-wt/DEU/GER29-14/2014/ G6P[9]	GER29-14	2014	Germany	G6	P9	I2	R2	C2	M2	A3	N2	T3	E2	H3
RVA/Human-wt/TUN/17237/2008/ G6P[9]	17237	2008	Tunisia	G6	P9	I2	R2	C2	M2	A3	N1	T6	E2	H3
RVA/Human/HUN/Hun7/1997/G6	Hun7	1997	Hungary	G6	P9	X	X	X	X	X	X	X	X	X
RVA/Human/-tc/USA/De584/1998/G6	De584	1998	USA	G6	P9	I2	R2	C2	M2	A3	N2	T1	E2	H3
RVA/Human-wt/BEL/B1711/2002/ G6P69]	B1711	2002	Belgium	G6	P6	I2	R2	C2	M2	A2	N2	T2	E2	H2
RVA/Human-tc/USA/DS-1/1976/ G2P[4]	DS-1	1976	USA	G2	P4	I2	R2	C2	M2	A2	N2	T2	E2	H2
Cattle														
RVA/Cow-tc/ USA/NCDV/ 1967/ G6P19]	NCDV	1967	USA	G6	P1	I2	R2	C2	M2	Ax	N2	T2	E6	Hx
RVA/Cow/JPN/AH1207/2022/ G6P[5]	AH1207	2022	Japan	G6	P5	I2	R2	C2	M2	A3	N2	T6	E2	H3
RVA/Cow/NorthIRE/R1WTA06/2013/G6P[5]	R1WTA06	2013	North Ireland	G6	P5	I2	R2	C2	M2	A3	N2	T2	E2	H3
RVA/Cow-tc/FRA/RF/1982/G G6P[1]	RF	1982	France	G6	P1	I2	R2	C2	M2	A3	N2	T3	E3	H3
Bat														
RVA/Bat-wt/ZMB/LUS12-14/2012/ G3P[3]	LUS12-14	2012	Zambia	G3	P3	I3	R2	C2	M3	A9	N2	T3	E2	H3

FeRVA = Feline rotavirus A.

### Sequence identification and genetic analysis

A pairwise comparison of the whole-genome sequences of Thai-FeRVAs with those of reference RVAs was performed. Our results showed that the *VP7* gene of Thai-FeRVA G3P[9] (CU32014) had high nucleotide and amino acid identities to Thai-FeRVA G3P[9] (THA/Meesuk/2021), previously reported in 2021 (99.7% nuclotide identities, 99.4% amino acid identities) and human RVAs from China (CHN/2020999/2020; CHN/23582009/2023) (98.4 ~ 98.7% nuclotide identities; 98.5 ~ 99.1% amino acid identities) (Table 5). For the Thai-FeRVA G6P[9] (CU33427), pairwise comparison of the *VP7* gene showed high nucleotide and amino acid identities to feline RVA from Japan (JPN/Mie20120017/2012) (98.1% nuclotide identities, 99.1% amino acid identities) and human RVA from Japan (JPN/KF17/2010) (98.4% nuclotide identities, 98.8% amino acid identities) (Table 6).

**Table 5:** Nucleotide (nt) and amino acid (aa) identities of the whole-genome of Thai-feline rotavirus A (FeRVA) G3P[9] (CU32014) with reference rotavirus A.

Strain	Genotype	Spp.	Year	Country	*VP7*	*VP4*	*VP6*	*VP1*	*VP2*	*VP3*	*NSP1*	*NSP2*	*NSP3*	*NSP4*	*NSP5*

					n (aa%)	n (aa%)	n (aa%)	n (aa%)	n (aa%)	n (aa%)	n (aa%)	n (aa%)	n (aa%)	n (aa%)	n (aa%)
RVA/Cat/THA/CU32014/2023	G3P[9]	Cat	2023	Thailand	100.0 (100.0)	100.0 (100.0)	100.0 (100.0)	100.0 (100.0)	100.0 (100.0)	100.0 (100.0)	100.0 (100.0)	100.0 (100.0)	100.0 (100.0)	100.0 (100.0)	100.0 (100.0)
RVA/Cat/THA/CU33427/2023	G6P[9]	Cat	2023	Thailand	71.2 (81.9)	97.4 (97.5)	97.0 (99.5)	90.7 (98.5)	87.7 (98.7)	78.4 (89.7)	97.2 (97.3)	97.1 (98.7)	98.4 (98.7)	97.5 (97.1)	98.1 (98.5)
RVA/Cat/THA/CU34812/2023	G6P[9]	Cat	2023	Thailand	71.3 (81.9)	96.1 (96.1)	97.0 (99.50)	90.6 (98.4)	90.2 (98.5)	78.2 (89.5)	97.2 (97.3)	96.9 (98.7)	98.4 (98.7)	97.5 (97.1)	98.1 (98.5)
RVA/Cat/JPN/Mie20120003f/2014	G6P[9]	Cat	2014	Japan	95.4 (97.2)	95.9 (96.0)	77.5 (94.8)	77.1 (95.0)	76.7 (96.3)	68.1 (80.5)	93.4 (94.1)	74.9 (85.8)	95.8 (98.4)	95.7 (97.1)	92.9 (92.7)
RVA/Cat/JPN/Mie20120017f/2014	G6P[9]	Cat	2014	Japan	71.7 (82.3)	95.9 (95.9)	93.4 (99.5)	82.7 (97.6)	87.0 (98.7)	90.6 (93.3)	96.3 (96.3)	98.2 (99.4)	96.8 (97.4)	96.9 (95.9)	97.8 (97.4)
RVA/Cat/THA/Meesuk/2012	G3P[9]	Cat	2012	Thailand	99.7 (99.4)	99.9 (99.9)	99.8 (99.7)	99.8 (99.7)	99.5 (99.7)	97.3 (97.6)	99.7 (99.2)	99.8 (99.4)	99.6 (99.0)	99.6 (99.4)	99.8 (100.0)
RVA/Cat/ITA/BA222/2005	G3P[9]	Cat	2005	Italy	98.6 (98.8)	96.5 (96.5)	93.1 (99.7)	90.1 (98.0)	87.5 (98.9)	91.0 (94.2)	96.9 (97.3)	79.4 (88.7)	98.4 (98.4)	72.6 (82.6)	98.0 (98.0)
RVA/Cat/AUS/Cat2/1964	G3P[9]	Cat	1964	Australia	94.9 (97.2)	95.1 (95.2)	76.9 (94.3)	77.1 (94.6)	77.2 (96.3)	69.6 (79.7)	91.0 (91.0)	79.3 (88.3)	71.1 (81.3)	82.5 (91)	95.1 (96.9)
RVA/Human/USA/3000381850/2017	G3P[9]	Human	2017	USA	97.2 (98.1)	96.4 (96.4)	93.4 (96.3)	90.9 (98.3)	87.8 (98.9)	89.4 (93.0)	97.3 (96.7)	97.0 (98.7)	97.8 (97.7)	72.6 (81.2)	97.8 (98.0)
RVA/Human/KOR/CAU-12-2-51/2012	G3P[9]	Human	2012	Korea	92.6 (95.3)	96.0 (96.1)	96.7 (99.5)	83.5 (97.6)	86.2 (98.9)	90.4 (93.1)	95.6 (94.9)	98.1 (99.0)	94.2 (96.4)	97.5 (96.5)	96.9 (96.4)
RVA/Human/KOR/CAU-14-1-262/2014	G3P[9]	Human	2014	Korea	95.0 (97.8)	94.3 (94.5)	77.3 (94.6)	77.0 (94.8)	76.8 (96.1)	69.5 (81.4)	90.9 (92.1)	75.6 (85.8)	72.0 (80.5)	90.8 (95.3)	88.7 (92.7)
RVA/Human/CHN/2020999/2020	G3P[9]	Human	2020	China	98.7 (99.1)	99.1 (99.4)	97.9 (99.7)	91.1 (98.4)	87.8 (98.6)	97.3 (97.4)	98.4 (98.4)	97.4 (98.1)	98.1 (98.7)	98.1 (97.1)	99.3 (99.5)
RVA/Human/CHN/23582009/2023	G3P[9]	Human	2023	China	98.4 (98.5)	99.0 (99.1)	97.8 (99.7)	90.7 (98.3)	87.6 (98.8)	97.0 (97.2)	98.1 (97.9)	97.4 (98.1)	98.1 (98.7)	98.9 (98.9)	99.3 (99.5)
RVA/Human-wt/GER-29-14/2014	G6P[9]	Human	2014	Germany	71.5 (82.3)	95.3 (95.4)	97.1 (99.7)	82.7 (97.7)	87.4 (99.3)	85.0 (92.9)	96.0 (96.0)	98.1 (99.4)	97.0 (97.4)	72.3 (84.6)	94.6 (96.4)
RVA/Human-wt/JPN/KF17/2010	G6P[9]	Human	2010	Japan	72.3 (82.6)	96.3 (96.4)	97.5 (99.7)	82.6 (97.7)	87.1 (99.1)	90.7 (93.4)	96.5 (97.1)	98.1 (99.4)	97.6 (98.0)	97.3 (96.5)	98.1 (98.5)
RVA/Human/JPN/AU-1/1982	G3P[9]	Human	1982	Japan	91.7 (96.2)	95.9 (96.0)	77.4 (94.3)	76.9 (95.0)	77.3 (96.1)	90.7 (80.8)	93.9 (95.4)	74.5 (86.1)	96.5 (98.0)	95.9 (97.1)	93.1 (92.7)
RVA/Human/JPN/FRV384/1994	G3P[3]	Human	1994	Japan	93.3 (95.6)	94.2 (94.3)	77.3 (94.0)	75.0 (94.0)	76.2 (96.4)	68.3 (79.9)	91.0 (91.0)	75.3 (85.4)	96.5 (98.4)	91.9 (95.3)	93.5 (93.7)
RVA/Dog/CHN/WH/2020	G3P[3]	Dog	2020	China	77.2 (89.0)	54.8 (59.0)	79.6 (95.3)	76.9 (94.9)	77.2 (95.9)	69.2 (79.9)	12.2 (0.5)	95.4 (97.1)	83.1 (92.6)	87.5 (92.3)	88.1 (91.6)
RVA/Dog/THA/CU25012/2020	G3P[3]	Dog	2020	Thailand	78.3 (89.0)	54.7 (58.9)	79.4 (95.3)	77.1 (95.1)	77.3 (96.3)	69.4 (80.0)	11.9 (1.0)	95.2 (97.4)	82.3 (93.3)	87.0 (92.3)	87.5 (91.0)

**Table 6:** Nucleotide (nt) and amino acid (aa) identities of the whole-genome of Thai-feline rotavirus A G6P[9] (CU33427) with reference rotavirus A (RVA).

Strain	Genotype	Spp.	Year	Country	*VP7*	*VP4*	*VP6*	*VP1*	*VP2*	*VP3*	*NSP1*	*NSP2*	*NSP3*	*NSP4*	*NSP5*

					n (aa%)	n (aa%)	n (aa%)	n (aa%)	n (aa%)	n (aa%)	n (aa%)	n (aa%)	n (aa%)	n (aa%)	n (aa%)
RVA/Cat/THA/CU33427/2023	G6P[9]	Cat	2023	Thailand	100.0 (100.0)	100.0 (100.0)	100.0 (100.0)	100.0 (100.0)	100.0 (100.0)	100.0 (100.0)	100.0 (100.0)	100.0 (100.0)	100.0 (100.0)	100.0 (100.0)	100.0 (100.0)
RVA/Cat/THA/CU34812/2023	G6P[9]	Cat	2023	Thailand	99.8 (100.0)	98.0 (98.0)	99.8 (100.0)	99.5 (99.9)	97.6 (99.8)	99.4 (99.6)	99.9 (100.0)	99.8 (100.0)	100.0 (100.0)	100.0 (100.0)	100.0 (100.0)
RVA/Cat/THA/CU32014/2023	G3P[9]	Cat	2023	Thailand	71.2 (81.9)	97.4 (97.5)	97.0 (99.5)	90.7 (98.4)	87.7 (98.7)	78.4 (89.7)	97.2 (97.3)	97.0 (98.7)	98.4 (98.7)	97.5 (97.1)	98.1 (98.5)
RVA/Cat/JPN/Mie20120003f/2014	G3P[9]	Cat	2014	Japan	71.7 (84.4)	95.3 (95.4)	77.2 (94.8)	76.4 (94.5)	75.8 (95.8)	69.6 (82.5)	94.3 (95.2)	76.4 (86.5)	96.8 (99.4)	95.5 (96.5)	93.8 (94.3)
RVA/Cat/JPN/Mie20120017f/2014	G6P[9]	Cat	2014	Japan	98.1 (99.1)	95.7 (95.7)	98.9 (99.5)	82.0 (97.8)	87.2 (98.9)	79.1 (90.4)	96.8 (96.7)	98.5 (99.4)	97.8 (98.4)	98.3 (97.7)	99.7 (99.0)
RVA/Cat/THA/Meesuk/2012	G3P[9]	Cat	2012	Thailand	71.3 (82.6)	96.2 (97.5)	97.0 (99.7)	90.8 (98.6)	87.8 (98.8)	80.0 (90.8)	97.3 (97.3)	97.3 (99.4)	98.6 (98.7)	97.5 (96.5)	98.3 (98.5)
RVA/Cat/ITA/BA222/2005	G3P[9]	Cat	2005	Italy	71.7 (82.6)	96.2 (86.3)	94.0 (99.7)	97.9 (98.8)	97.3 (99.5)	80.1 (90.8)	97.3 (97.9)	79.5 (89.7)	99.1 (99.4)	72.3 (83.2)	99.8 (99.5)
RVA/Cat/AUS/Cat2/1964	G3P[9]	Cat	1964	Australia	71.7 (84.8)	94.4 (94.5)	77.5 (94.3)	77.2 (93.9)	76.3 (95.8)	69.4 (82.1)	91.8 (91.9)	79.3 (89.0)	70.8 (81.3)	82.7 (92.3)	95.6 (97.4)
RVA/Human/USA/3000381850/2017	G3P[9]	Human	2017	USA	71.3 (82.3)	97.3 (97.3)	94.3 (96.3)	98.8 (99.4)	98.6 (99.8)	79.2 (90.4)	98.3 (97.5)	99.3 (100.0)	98.6 (98.7)	72.6 (81.9)	99.7 (99.5)
RVA/Human/KOR/CAU-12-2-51/2012	G3P[9]	Human	2012	Korea	72.9 (84.4)	95.7 (95.8)	97.7 (99.5)	83.0 (97.6)	89.7 (98.9)	79.4 (90.3)	96.3 (95.8)	98.0 (99.0)	94.9 (97.4)	97.7 (97.1)	98.8 (98.0)
RVA/Human/KOR/CAU-14-1-262/2014	G3P[9]	Human	2014	Korea	72.0 (84.4)	93.7 (93.9)	77.6 (94.6)	76.6 (94.4)	75.6 (95.6)	70.7 (82.8)	92.0 (93.4)	76.4 (86.1)	71.7 (80.9)	90.8 (94.7)	89.5 (92.1)
RVA/Human/CHN/2020999/2020	G3P[9]	Human	2020	China	71.7 (82.6)	97.2 (95.6)	98.1 (99.7)	99.0 (99.6)	96.3 (99.3)	79.9 (91.3)	97.8 (98.6)	98.0 (99.4)	97.8 (98.7)	97.5 (96.5)	98.8 (99.0)
RVA/Human/CHN/23582009/2023	G3P[9]	Human	2023	China	71.3 (81.9)	97.4 (97.4)	98.0 (99.7)	98.7 (99.5)	96.1 (99.5)	79.8 (91.0)	97.6 (98.1)	98.0 (99.4)	97.8 (98.7)	98.3 (98.3)	98.8 (99.0)
RVA/Human-wt/GER-29-14/2014	G6P[9]	Human	2014	Germany	98.9 (99.1)	95.1 (95.2)	98.7 (99.7)	81.9 (97.9)	88.1 (98.8)	80.0 (92.0)	96.6 (96.7)	98.0 (99.4)	97.8 (98.4)	72.6 (83.9)	95.1 (96.9)
RVA/Human-wt/JPN/KF17/2010	G6P[9]	Human	2010	Japan	98.4 (98.8)	96.0 (96.1)	99.0 (99.7)	82.0 (97.9)	87.6 (99.1)	79.1 (90.4)	96.6 (97.5)	98.0 (99.4)	98.4 (99.0)	98.7 (86.3)	100.0 (100.0)
RVA/Human/JPN/AU-1/1982	G3P[9]	Human	1982	Japan	70.5 (84.8)	95.1 (95.2)	77.5 (94.3)	76.1 (94.3)	76.3 (95.7)	70.7 (82.8)	94.6 (96.4)	75.5 (86.9)	97.0 (99.0)	96.1 (97.7)	94.0 (94.3)
RVA/Human/JPN/FRV384/1994	G3P[3]	Human	1994	Japan	72.9 (84.8)	93.4 (93.6)	77.5 (94.0)	75.3 (93.7)	75.5 (96)	69.0 (81.8)	91.6 (92.1)	76.1 (85.8)	96.6 (99.4)	91.4 (94.7)	94.4 (95.3)
RVA/Dog/CHN/WH/2020	G3P[3]	Dog	2020	China	71.9 (86.2)	55.1 (59.2)	79.6 (95.3)	76.4 (94.3)	76.5 (95.5)	70.0 (82.2)	12.4 (0.5)	95.0 (97.8)	83.2 (93.3)	87.9 (93.5)	89.3 (92.1)
RVA/Dog/THA/CU25012/2020	G3P[3]	Dog	2020	Thailand	72.2 (86.6)	55.0 (59.1)	79.9 (95.3)	76.5 (94.4)	76.5 (95.8)	70.2 (82.4)	12.2 (1.0)	94.8 (98.1)	82.4 (94.0)	88.0 (93.5)	89.1 (91.6)

FeRVA = Feline rotavirus A.

Genetic analysis of the VP7 antigenic region was conducted by comparing the amino acid sequences of Thai-FeRVAs and reference RVAs from various genotypes and host species. Analysis of VP7 antigenic regions A, B, C, and F showed no significant amino acid substitutions (no mutations) in FeRVA G3P[9] compared to the previous Thai-FeRVA (THA/Meesuk/2021) and other closely related RVAs. Similarly, Thai-FeRVA G6P[9] had unique amino acids in regions A, B, C, and F, matching those found in FeRVA from Japan (JPN/Mie20120017/2012) and human RVA from Japan (JPN/KF17/2010). One amino acid substitution (S90P) was noted at position 90 in the Thai-FeRVA G6P[9] (Table 7).

**Table 7:** Genetic analysis of the VP7 genes of Thai-feline rotavirus A and reference rotavirus A (RVA) from dogs, cats, cattle, bats, and humans.

Viruses	Species	Country	Year	Genotype	Lineage	Glycosylation site		Amino acid position (*VP7* gene)													

								Unique amino acid position in the A region						Unique amino acid position in the B region			Unique amino acid position in C region			Unique amino acid position in F region	

						69-71	238-240	90	91	94	96	97	100	146	147	149	212	217	221	241	242
Reference strains of RVA																					
Mie20120017f	Cat	JPN	2012	G6	I	NST	NVT	S	N	A	T	E	N	S	A	E	P	T	T	I	E
KF17	Human	JPN	2010	G6	I	NST	NVT	S	N	A	T	E	N	S	A	E	P	T	T	I	E
GER29-14	Human	DEU	2014	G6	I	NST	NVT	S	N	A	T	E	N	S	A	E	P	T	T	I	E
Hun7	Human	HUN	1997	G6	I	NST	NVT	S	N	A	T	E	N	S	A	E	P	T	T	I	E
Hun3	Human	HUN	1997	G6	II	NST	NVT	S	N	A	T	E	N	S	A	E	P	T	T	I	E
Hun4	Human	HUN	1997	G6	II	NST	NVT	S	N	A	T	E	N	S	A	E	P	T	T	I	E
10733	Buffalo	ITA	2003	G6	II	NST	NVT	S	N	A	T	E	N	S	T	E	P	T	T	T	E
Hun-BoRo4	Bovine	HUN	2010	G6	II	NST	NVT	S	N	A	T	E	N	S	T	E	P	T	T	T	E
RF	Bovine	FRA	1982	G6	III	DST	NVT	T	N	A	T	E	N	S	T	E	P	T	T	T	.A
CU25012	Dog	THA	2020	G3	b2	NST	DVT	A	T	N	N	S	D	A	A	Q	V	E	T	T	T
WH	Dog	CHN	2020	G3	b2	NST	DVT	T	T	N	N	S	D	A	A	Q	V	E	T	T	T
BA222	Cat	ITA	2005	G3	a	NST	NVT	A	T	N	N	S	D	A	A	Q	S	E	A	T	N
FRV384	Cat	JPN	1994	G3	a	NST	NVT	A	T	N	N	S	N	A	A	Q	S	E	A	T	N
Meesuk	Cat	THA	2021	G3	a	NST	NVT	A	T	N	N	S	D	A	T	Q	S	E	A	T	N
Cat2	Cat	AUS	1984	G3	a	NST	NVT	A	T	N	N	S	D	A	T	Q	S	E	A	T	N
Mie20120003f	Cat	JPN	2012	G3	a	NST	NVT	A	T	N	N	S	D	A	T	Q	S	E	A	T	N
Mie20120016f	Cat	JPN	2012	G3	a	NST	NVT	A	T	N	N	S	D	A	T	Q	S	E	A	T	N
3000381850	Human	USA	2017	G3	a	NST	NVT	A	T	N	N	S	D	A	T	Q	S	E	A	T	N
CAU12-2-51	Human	KOR	2012	G3	a	NST	NVT	A	T	N	N	S	D	A	A	Q	S	E	A	T	N
CAU-14-1-262	Human	KOR	2014	G3	a	NST	NVT	A	T	N	N	S	D	A	T	Q	S	E	A	T	N
AU-1	Human	JPN	1982	G3	a	NST	NVT	A	T	N	N	S	D	A	T	Q	S	E	A	T	N
2020999	Human	CHN	2020	G3	a	NST	NVT	A	T	N	N	S	D	A	T	Q	S	E	A	T	N
23582009	Human	CHN	2020	G3	a	NST	NVT	A	T	N	N	S	D	A	T	Q	S	E	A	T	N
This study																					
CU33427	Cat	THA	2023	G6	I	NST	NVT	P	N	A	T	E	N	S	A	E	P	T	T	I	E
CU34812	Cat	THA	2023	G6	I	NST	NVT	P	N	A	T	E	N	S	A	E	P	T	T	I	E
CU32014	Cat	THA	2023	G3	a	NST	NVT	A	T	N	N	S	D	A	T	Q	S	E	A	T	N

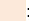
 : Represent RVA genotype G6.

## DISCUSSION

### Overview of RVA in cats

RVA is a significant viral pathogen that causes enteric infections in various animal species, including cats. This virus mainly causes diarrhea and leads to dehydration, weight loss, and death, especially in young cats. RV mainly spreads through the fecal-oral route via contaminated water, food, or surfaces, emphasizing the need for effective control and prevention measures.

### Prevalence of FeRVA in the present study

In this study, we conducted a cross-sectional survey of FeRVAs in Thailand from January 2022 to December 2023. A total of 636 rectal swab samples were tested for RVA by RT-PCR targeting the *NSP5* gene. The results indicated that RVA positivity was 1.41% (9/636). Compared with earlier studies in Thailand, the occurrence of FeRVA positivity was higher than in the previous report [14], but lower than in other countries, such as UK [5], Japan [6], Australia [18], Brazil [19], and Germany [20]. This variation may be due to differences in geographic regions, sampling sites, animal selection, and disease control strategies. Although the positivity rate in our samples was low, our findings are consistent with prior RVA surveillance studies in cats [5, 14]. Such low prevalence is often seen in community or shelter populations, where multiple factors, such as timing of sample collection, intermittent viral shedding, subclinical or asymptomatic infections, and geographic or seasonal variations, may influence RVA transmission.

### Seasonal and clinical distribution of FeRVA

In this study, the highest FeRVA positivity was observed in cats during winter, suggesting that the local winter environment may promote viral transmission [21]. However, this finding did not match the results from the UK study, where RVAs were significantly detected in the summer, when several kittens were present [5]. FeRVA positivity was high in asymptomatic cats, indicating that FeRVA infection can occur in both symptomatic and asymptomatic cases, although diarrhea remains a major clinical sign in cats. Our findings are consistent with previous reports from the UK [5] and Brazil [19], which found no clear differences among RVA genotypes in clinical signs in cats.

Vaccines targeting feline or canine RVA are rarely used in Thailand. Commercial vaccines are typically developed for genotypes from other regions and may provide limited or no cross-protection against local FeRVAs. The lack of a history of genotype-specific vaccination and the absence of local vaccination protocols hinder our ability to accurately assess the impact of prior immunity on susceptibility or asymptomatic carriage of RVAs in this feline population.

### Age-related trends and epidemiological associations

The high positivity for FeRVA was observed at a young age in the cats in this study, consistent with findings from studies in studies in UK [5], Thailand [14], and the Brazil [19, 22]. Overall, the links between FeRVA positivity and the animals’ age, clinical status, and season were not statistically significant. This result was due to several connected factors, including the low overall positivity rate, consistent clinical signs, and small sample size. These factors may have affected epidemiological patterns, as noted in previous single-center studies [5, 14, 19]. This limitation emphasizes the need for larger or multicenter studies to better understand the risk factors for FeRVA infection.

### Phylogenetic characteristics of G3P[9] FeRVA

Phylogenetic analysis of the *VP7* and *VP4* genes identified one Thai-FeRVA genotype G3P[9] (CU32014). Whole-genome analysis showed that the Thai-FeRVA genotype G3P[9] was closely related to the FeRVA genotype previously characterized in Thailand (THA/Meesuk/2021) and human RVAs from China (CHN/2020999/2020 and CHN/23582009/2023). The Thai-FeRVA G3P[9] also exhibited a genetic constellation similar to that of the FeRVA previously reported in Thailand and human RVAs from China and Korea. This suggests potential interspecies transmission of the G3P[9] genotype between cats and humans, possibly involving reassortment events between feline and human RVs, as previously documented [7, 23].

Furthermore, Thai-FeRVA G3P[9] was detected in clinically healthy cats, whereas a previous study reported the presence of Thai-FeRVA G3P[9] in a cat with bloody diarrhea [7]. Therefore, the FeRVA G3P[9] genotype can be found in both asymptomatic and symptomatic animals. It can also be assumed that the FeRVA G3P[9] genotype is still circulating as the second most common genotype in cat populations in Thailand, following the earlier report of the FeRVA G3P[3] genotype [14].

### Phylogenetic characteristics of G6P[9] FeRVA

Phylogenetic analysis of *VP7* and *VP4* genes identified two Thai-FeRVA genotypes, G6P[9] (CU33427 and CU34812). To our knowledge, this is the first report of novel FeRVA genotypes in cat populations in Thailand. The WGS of these two Thai-FeRVA genotypes G6P[9] showed close relation to feline (JPN/Mie20120017/2012) and human (JPN/KF17/2010) RVA strains from Japan. Additionally, these Thai-FeRVAs G6P[9] shared a genetic constellation similar to that of feline and human RVAs from Japan. Based on this genetic constellation, the Thai-FeRVA genotype G6P[9] may have the potential for interspecies transmission [6] and could have spread within the cat population in Thailand. Furthermore, the Thai-FeRVAs G6P[9] were found in asymptomatic cats, consistent with a recent report from Japan [6, 24].

### VP7 Antigenic region analysis

Genetic analysis of the VP7 antigenic regions (A, B, C, and F) showed that Thai-FeRVA G3P[9] and G6P[9] were identical to other G3 and G6 RVAs found in dogs, cats, and humans, and matched findings from previous studies [25]. The examination of antigenic regions A, B, C, and F revealed conserved amino acid determinants across RVAs from animals and humans, indicating host-specific diversification and multiple zoonotic reassortment events, especially between bovine and human RV [25–27].

Notably, the amino acid substitution at position 90 in Thai-FeRVA G6P[9] may be a unique change among Thai viruses. However, since only one amino acid substitution (S90P) was observed, this does not clearly indicate significant genetic diversity of the *VP7* gene among reference RVAs from cattle, buffalo, and humans. The VP7 protein is a major outer capsid glycoprotein that is crucial for eliciting neutralizing antibody responses [28, 29]. Hence, antigenic variation in VP7 can affect host immune recognition and vaccine effectiveness. For instance, previous studies have shown that amino acid changes in VP7 can enable immune evasion and the virus’s adaptation to a new host [29, 30]. Therefore, ongoing molecular surveillance and functional analysis of these mutations are essential to better understand their potential impact on host specificity, immune escape, and cross-species transmission risks.

### Origin and potential transmission pathways of Thai G6P[9] FeRVA

Phylogenetic analysis and genetic constellation of Thai-FeRVA G6P[9] revealed a close relationship with both FeRVA and human RVA strains from Japan. The origin of Thai-FeRVA G6P[9] may be linked to the import or movement of animals through international travel. Genetic reassortment between RVA strains from different host species may have occurred. Although there is currently no evidence of FeRVA cross-transmission from cats to humans in Thailand, the possibility of genetic reassortment and interspecies transmission events cannot be ruled out due to the lack of such reports. Comprehensive investigations involving both human and feline populations are essential to examine potential cross-species transmission and to determine the origins of FeRVA G6P[9] in the country.

### Public health significance and global comparison

So far, 15 complete FeRVA genome sequences have been submitted to GenBank. Additionally, the FeRVA genotypes G3P[3], G3P[9], and G6P[9] have been reported, indicating their possible interspecies transmission from cats to humans in some Asian countries, such as Japan [6, 9], Korea [10], Thailand [7, 14], and in some European countries, such as Italy [11] and the UK [5]. Hence, the detection of the FeRVA G3P[9] and G6P[9] genotypes in this study underscores the potential for transmission between cats and humans in Thailand.

### Study limitations

This study has several limitations. For example, it used a cross-sectional sample collection, which may not represent the broader cat population. The absence of longitudinal data also limits the ability to assess persistent or recurring infections. Additionally, incomplete clinical histories of the cats prevented the identification of potential correlations between infection status and health outcomes. Moreover, some other risk factors that could influence transmission dynamics, such as cohabitation with other animals, household hygiene, or dietary habits, were not recorded. Finally, there have been no human cases of RVA G6P[9] in Thailand to confirm zoonotic transmission [31]. The zoonotic potential of the Thai-FeRVA G6P[9] was inferred solely on the basis of genetic similarity. These limitations underscore the need for more comprehensive surveillance and larger sampling.

## CONCLUSION

This study offers the first comprehensive molecular and whole-genome analysis of FeRVA circulating in domestic cats in Thailand. The overall FeRVA positivity was low (1.41%), but the detection of both G3P[9] and the novel G6P[9] genotypes shows that genetically diverse RVA strains continue to circulate among cats. WGS revealed that the Thai G3P[9] strain was closely related to previously reported Thai-FeRVA and human strains from China, whereas the G6P[9] strains showed strong genetic similarity to feline and human RVA strains from Japan. These results highlight the potential for cross-species transmission and suggest ongoing viral movement and possible reassortment between feline and human hosts. VP7 antigenic region analysis further confirmed conserved amino acid motifs among Thai strains and their global counterparts, with only one unique substitution (S90P) identified in G6P[9].

From a practical perspective, detecting FeRVA in asymptomatic cats highlights the importance of surveillance programs that include both healthy and clinically affected animals, since silent carriers may help maintain the virus within the population. The genomic similarities between Thai and international strains also suggest that cross-border animal movement, lack of targeted vaccination programs, and shared environments could promote viral spread. These findings support the need for enhanced One Health–based monitoring to prevent unrecognized transmission risks, especially in multi-species settings, veterinary clinics, and urban areas.

A key strength of this study is the use of WGS, which enabled precise genotype identification, genetic constellation profiling, and detailed phylogenetic analysis. The study also offers updated epidemiological data from Thailand, filling a significant knowledge gap. However, some limitations remain. The cross-sectional design limits the ability to track persistence or reinfection; clinical histories were incomplete for some animals, and additional risk factors, such as cohabitation or environmental hygiene, were not assessed. Furthermore, the zoonotic transmission potential of G6P[9] could not be confirmed because there have been no human RVA G6P[9] cases in Thailand.

Future research should incorporate longitudinal and multicenter surveillance, parallel studies in human and animal populations, and functional analyses to clarify the biological importance of observed genomic variations. Expanding sampling across different provinces and environments, along with environmental and metagenomic studies, would enhance understanding of transmission pathways and viral evolution.

In conclusion, this study broadens the current understanding of FeRVA epidemiology and genetic diversity in Thailand and stresses the importance of ongoing genomic surveillance. The detection of both G3P[9] and the emerging G6P[9] genotypes underscores the need for integrated One Health strategies to identify, monitor, and reduce potential interspecies transmission risks between cats and humans.

## DATA AVAILABILITY

The authors state that the data supporting this study’s findings are available in the supplemental tables. The nucleotide sequence data supporting the findings are publicly accessible in the GenBank database at https://www.ncbi.nlm.nih.gov/genbank/, under accession numbers PV650015-47.

## AUTHORS’ CONTRIBUTIONS

YNT, KC, EMP, and HWP: Collected the samples. YNT, CN, EC, WJ, and SC: Conducted virus detection, whole-genome analysis, and phylogenetic studies. KC, CN, SC, and SP: Contributed to the phylogenetic analysis. YNT: Drafted the manuscript. AA: Designed the study, analyzed the data, and drafted and revised the manuscript. All authors have read and approved the final version of the manuscript.
